# International workshop on the future of community child healthcare

**DOI:** 10.1186/s13584-019-0355-z

**Published:** 2019-12-05

**Authors:** Zachi Grossman, Boaz Porter, Joseph Meyerovitch, Lisa Rubin, Jacob Kuint, Efrat Wechsler, Doron Dushnitzky, Shai Ashkenazi

**Affiliations:** 1 Pediatric clinic, Maccabi Healthcare Services, 26 Rofe Hamachtarot, 69372 Tel Aviv, Israel; 2 Faculty of Health Sciences, Ben Gurion University of the Negev, Be’er Sheva, Israel; 3 Clalit Health Services and Schneider Children’s Medical Center, Petah Tikva, Israel; 4 Sackler faculty of medicine, Tel Aviv University, Tel Aviv, Israel; 5 Ministry of Health, Jerusalem, Israel; 6 School of Public Health, University of Haifa, Haifa, Israel; 7 K.S.M Research and innovation Institute, Maccabi Healthcare Services, Tel Aviv, Israel; 8 Meuhedet Health Organization, Tel Aviv, Israel; 9 Leumit Health Services, Tel Aviv, Israel; 10 Tel Aviv University, Tel Aviv, Israel; 11 Adelson School of Medicine, Ariel University, Ariel, Israel

**Keywords:** Community pediatrics, Psychosocial morbidity, Residency training, Integrated care

## Abstract

Increasing numbers of children with developmental, emotional, and psychosocial issues require adaptation of the services provided by pediatricians in the community. An international workshop that took place in Israel on June 3–4, 2019, addressed this need. Local policy makers and international experts discussed the following topics: (1) the future of training in community pediatrics; (2) enhancing the prestige of the community pediatrician; (3) development of management and research skills; (4) academic advancement within community pediatrics; (5) the future content of community pediatric practice; (6) visit length and community pediatricians’ reimbursement; (7) developing the collaborative model of care in community pediatrics and (8) integrating child healthcare. The meeting provided a venue to understand the challenges and to formulate recommendations to policymakers. A key target highlighted was the increased exposure of all pediatric residents to community pediatrics. This gained the support of the Chief Executive Officers of all four Health Funds in Israel. This document provides a synopsis of the topics addressed and suggested recommendations.

## Introduction

In 1975, Robert Haggerty, a pediatrician from Rochester in the US, originated the term “the New Morbidity” [1] to reflect the importance of the developmental, emotional and psychosocial issues of children in pediatric practice. The American Academy of Pediatrics (AAP) adopted this concept [2, 3]. The burden of problems in one of these areas has increased dramatically in recent decades [4]. According to various estimates, over 20% of the children who attend the pediatrician’s community pediatric clinic for any reason suffer from at least one of the problems [5].

According to a recent policy statement by the AAP, this pattern of morbidity requires adaptation of child healthcare services provided by pediatricians in the community [6]. Pediatric residents must have exposure to this morbidity through rotations pediatric community clinics, so that they may acquire the knowledge and tools to enable them to best manage psychosocial morbidities.

Addressing these issues is time consuming. Service provision needs to be redesigned both in terms of time allotted to visits and reimbursement. Effective delivery of services requires also requires creating collaborative interfaces. Partnership with other stakeholders of child health, such as the education and welfare systems, is most likely to positively impact on the management of these children.

The workshop, which took place on June 3–4, 2019, is the first to address the need to redesign community child healthcare in Israel. It was organized by the Israel National Institute for Health Policy Research (NIHPR) [7]. The NIHPR periodically organizes international workshops on current health topics and is instrumental in bringing both national and international experts together for serious discourse on burning important issues. The NIHPR also the sponsors of the International Journal of Health Policy Research, in which this meeting report is being published.

Sixty-two key policymakers and opinion leaders participated in the workshop. They represented the Ministry of Health, the four Health Funds – Clalit, Maccabi, Meuhedet and Leumit, pediatric professional organizations (Israel Pediatric Association, the Israel Ambulatory Pediatric Association and the Israel Clinical Pediatric Association) Goshen (a non-profit organization for dedicated to professional psychosocial and developmental education), the Israel National Council for the Young Child and the Israel Scientific Council. The Scientific Council is the scientific arm of the Israeli Medical Association. The Council is responsible, under the Physicians Ordinance, for the planning and supervision of the physician specialization system in Israel and for continuing education programs in medicine [8].

The event, which is described below, provided an exceptional opportunity to tackle crucial and little discussed aspects of this care.

The topics addressed at the meeting included:

- The future of training in community pediatrics
- Increasing the prestige of the discipline of community pediatrics
- Future content of community pediatric practice
- Management of the future community pediatric practice including issues of visit length and reimbursement
- Research and academic recognition in community pediatrics
- Developing the collaborative model of care in community pediatrics
- Integrated child healthcare

## Synopsis

The program was opened by Orly Manor, Chair of the Board of Directors of the National Institute for Health Policy Research (NIHPR) and Zamir Halpern, the Scientific Director of the institute. Prof. Manor introduced the NIHPR to the workshop participants, stressing its role as the main funding body for research in healthcare services. NIHPR workshops and conferences are held to encourage discussion of key health policy issues. Prof. Halpern stated his belief that the health of a nation can be judged by the health of its youngest members and pointed out that “children are not small adults”. He pointed out that the changing needs of child healthcare and the high burden of chronic conditions can be managed only by the integration of clinical practice with public health actions. Prof. Eitan Kerem, the Chairman of Goshen and the Chairman of Pediatrics in Hadassah Hospital in Jerusalem, highlighted the importance of investing in young children but noted the current lack of interest and monetary investments by relevant stakeholders. Facing the current fragmentation of services, he stressed the importance of creating integrated care for children.

The first keynote lecture was given by Prof. Frank Oberklaid, the director of the Centre for Community Child Health in Melbourne, Australia. He addressed the challenges in children’s health and wellbeing, focusing on the leadership role of pediatricians. He described the increasing prevalence of psychosocial and behavioral issues. In Australia, autism, attention deficit hyperactivity disorder (ADHD) and sleep disturbances are the most frequent diagnoses noted for children consulting a pediatrician [9]. Early adversity results in high stress levels, and thus poverty and social disadvantage in early years have a major impact on child development and psychosocial wellbeing. Early intervention is critical. Facing the current fragmentation of services, Prof. Oberklaid called for a leadership role for pediatricians, a role that will include the following elements: focusing on prevention and early intervention, reorganizing of community services, addressing equity issues in child health and establishing an advocacy role for pediatricians.

### The future of training

Prof. Shai Ashkenazi, the Dean of the Adelson Medical School at Ariel University and Chairman of the Israel pediatric Association opened the session. He emphasized the role of medical schools in teaching future physicians about community pediatrics. Israeli medical students spend significant time in hospital-based ambulatory pediatric clinics. However, they have inadequate and unequal exposure to community-based pediatric clinics: In Hebrew University-Hadassah Medical School, there is no rotation dedicated to community pediatrics exposure. In Tel Aviv University, every student does a one-week rotation in a community pediatric clinic in the sixth year. In the Technion, Ben Gurion University and Bar Ilan, there is a three-day, two weeks and one-week community pediatrics exposure, respectively. The newly launched medical school in Ariel University plans to have a three-week rotation in community pediatrics. The reasons for the limited exposure afforded medical students include the very short time allocated for patient encounters in community clinics, inadequate space for hands-on examination by the students, shortage in the community of certified pediatricians, lack of subacute or chronic patients and the absence of compensation to the pediatrician for teaching activities. The above barriers can be overcome by joint efforts to create a new atmosphere among all stakeholders (dean, faculty, community clinics) that acknowledges the importance of exposure to the community.

In Israel, there is currently little exposure of pediatric residents to community pediatrics during their residency, and therefore the knowledge and skills necessary for handling the new morbidity issues is often inadequate [10, 11]. Prof. Jacob Urkin, from Ben Gurion University and Clalit Health Services spoke about graduate and post-graduate training in community pediatrics. He stressed the important elements in the process of training in the community: the curriculum that was developed for that purpose, the faculty development program to instruct the teachers, advanced optimal teaching methodologies, the need to adapt the teaching methods to the community set-up and the establishment of a proper evaluation of the training.

Dr. Michael Davidovitch, the head of the child development and ADHD section in Maccabi healthcare services focused on the missing pieces in community pediatrics training, developmental, and neurobehavioral pediatrics. He presented data showing a dramatic increase in the prevalence of autistic spectrum disorder and ADHD in the recent decade [12]. He pointed out that the 3-year pediatric residency programs in the US require at least a 1 month of rotation in child development centers for exposure to developmental and behavioral pediatrics [13]. In comparison, the post- graduate training in Israel lasts 5.5 years, but there is no mandatory learning and exposure to child developmental disabilities. Dr. Davidovitch concluded by recommending the following: teaching pediatricians in training about normal and abnormal development, thus providing them with appropriate expertise to deal with questions of development and behavior, teaching the use of developmental screening tools such as Ages & Stages Questionnaires (ASQ), and providing ongoing training in the management and coordination of for children with complex health conditions.

The session ended with a panel discussion focusing on the need to expose all pediatric residents to community pediatrics. The panel was moderated by Dr. Shimon Barak, the president of the Israel Ambulatory Pediatric Association. The participants were Prof. Gidi Paret, the chair of the scientific council of the Israel Medical Association, Prof. Shai Ashkenazi, the president of the Israel Pediatric Association, and four HMO representatives – Dr. Shlomit Yaron (Clalit), Dr. Naomi Siegal (Maccabi), Dr. Rinat Cohen (Meuhedet) and Dr. Refael Cayam (Leumit). Participants agreed that exposing all pediatric residents to community pediatrics is a must and this should be reflected in the curriculum. Some called to examine the idea of a one-day weekly continuity clinic instead of a block rotation. The need to bolster the prestige of community pediatrics in the eyes of the residents was noted to be of extreme importance, and this could be accomplished by establishing community pediatrics as a recognized subspecialty. In order to succeed, the obligatory increased exposure approach should be accompanied by means to ensure a quality exposure experience. Participating pediatricians need to be compensated for teaching. Panel participants believe, wherever possible, group practices should be preferred venues for teaching rather than solo practices. However, not all in the audience agreed with this statement.

### Empowering the future community pediatrician – how to create prestige?

The lack of exposure of residents to community pediatrics causes this part of the profession to be perceived as dull and boring. The absence of opportunities for research, teaching and academic promotion, aspects that enhance the prestige in hospital pediatrics contributes to community pediatrics poor image. As a result, at the end of their training, most graduating residents do not want to be fully employed in the community, despite the high salaries offered by the health funds. This may explain the shortage of pediatricians in the community.

The session was opened by Prof. Richard Wasserman, former director of The Pediatric Research in Office Setting (PROS) affiliated with the AAP, who gave a lecture on how research by community pediatricians can generate new knowledge, better patient care, and build a pediatric career. He described several models of participation of pediatric practitioners in research activities. Community pediatricians’ motives for participating in research activity include curiosity as to the effectiveness of their work, altruism (contributing through their own efforts to scientific advance) and a desire for affiliation (i.e., being a part of something larger). Community-based research contributes to better patient care as guidelines are updated to reflect new knowledge from research in practice and practitioners are more likely to follow guidelines based on research from practice settings similar to their own. Significantly, a recent survey demonstrated that engagement in research activity was associated with decreased burnout among primary care pediatricians in Israel [14].

The situation of current and future pediatric workforce in Israel was discussed by Lisa Rubin, the Head of the Department of Maternal and Child Health in the Ministry of Health (MOH). Israel has the highest fertility rate among OECD countries, 3.08 children per mother, with almost 3 million children aged 0–17 years. The Ministry of Health reports periodically on the number of physicians licensed and awarded specialty recognition. The number of pediatricians increased by 135% from 1000 in 1990 to 2362 in 2018, and pediatrics is the second most prevalent specialty in Israel [15]. The rate of pediatricians per 1000 population and per 1000 children aged 0–14 years is 0.24 and 0.79 respectively [16, 17]. The number of pediatric residents increased from 508 in 2012 to 645 in 2017. Women comprise 53% of pediatricians, compared to only 37% in 1990. There is however no accurate information on the actual number of practicing pediatricians in the country to provide care for the growing number of children. Dr. Rubin highlighted the necessity of collaboration with other professionals, agencies and the parents, and urged to change focus from the individual child to all children in the community.

The last part of the session was a lecture on the need to redefine, rethink and rebrand community pediatrics as a prestigious career pathway. The speaker, Dr. Hava Gadassi, medical director of Goshen, highlighted the additional roles that should become an integral part of the future community pediatrician: being academic, having the capacity to be a director and become influential in the child’s community. This transformation is the key to creating a prestigious career that will attract young residents to choose community pediatrics as a career. The session ended in a panel discussion on the new roles - management, research and academic activities - in community pediatrics. The moderator was Dr. Manuel Katz, president of Goshen, and the participants were representatives of the 4 Health Funds – Prof. Avner Cohen and Prof. Joseph Meyerovitch (Clalit), Prof. Jacob Kuint (Maccabi), Dr. Efrat Wechsler (Meuhedet) and Dr. Doron Dushnitzki (Leumit). Each participant from the Health Funds described the various current career-promoting initiatives in their respective organizations. The participants conferred that most pediatricians prefer to be only to be clinical service providers, and at the outset are concerned primarily in the salaries offered. Therefore, to ensure the success of career developing initiatives, the critical elements of coaching, role modeling (for management), and ensuring protected time and infrastructure for research are needed.

### The future content of community pediatric practice

Dr. Deena Zimmerman, Supervising Maternal Child Physician in the Jerusalem District of the MOH, opened the session by describing the role of maternal child health clinics (MCHCs) - Tipot Halav- in delivering preventive pediatric care to children 0–6 years. She emphasized the importance of Israel’s literature-based well childcare guidelines, which were recently published [18] and alerted the audience to the general physician shortage in MCHCs, and the specific instance of successful recruitment of physicians to MCHCs in Jerusalem.

The preliminary results of two new original studies were then discussed by Yael Ashkenazi, a researcher from the Brookdale Institute, and Prof. Boaz Porter from Goshen. The two studies focused on the under-performance of screening and psychosocial interventions by community pediatricians. The first study was a qualitative study of 21 semi-structured interviews with child development institute (CDI) directors that aimed to understand their perspective regarding the role of primary care pediatricians in the field of child development (CD). The directors interviewed thought that most primary care pediatricians (PCPs) have little involvement in CD issues. They would like to see more involvement, but they noted the systemic barriers of lack of time, appropriate compensation and knowledge, that all require system level responses. The second study focused on the physician role in mental health (MH) from the pediatricians’ and parents’ perspectives. The study concluded that pediatricians’ training and practice do not reflect MH needs in the community. Limited training is associated with limited interest and limited involvement, and training plays a role in overcoming the barriers of time, knowledge, competence, confidence and professional identity.

The concluding lecture in the session focused on chronic disease management by the community pediatrician. Prof. Joseph Meyerovitch noted that in Clalit Health Services (CHS), 27.1% of children age 0–18 are affected by at least one chronic condition. He presented a population-based study showing that pediatric patients with type 2 diabetes could achieve reasonable glycemic control in both community and outpatient hospital settings [19]. What can be done to optimize the management of chronic diseases in the community pediatric clinic? Prof. Meyerovitch suggested: improving medical education; mandatory resident rotations in an ambulatory clinic; mandatory resident rotations in a child development center; use of a comprehensive data system in combination with focused organization policy and tools; building team work with nurses and administrative personnel combined with protocols and computerized tools; on-line medicine, on-line access to information and treatment algorithms; and increasing the number of Pediatric Health Centers.

The first day of the workshop ended with a panel discussion on visit length and pediatricians’ reimbursement. The moderator was Prof. Gabi Bin Nun from the Department of Health Systems Management in Ben Gurion University. The participants were 4 Health Funds’ representatives: Dr. Shlomit Yaron (Clalit), Prof. Nachman Ash (Maccabi), Dr. David Dvir (Meuhedet) and Prof. Shlomo Vinker (Leumit). The participants agreed that the current financial model of reimbursement does not meet current needs and should be redesigned. Competition between the Health Funds drives their immediate interest to provide maximal availability for acute care pediatric conditions. Thus, the main reimbursement incentive is volume-based. The resulting short visit times make it difficult to properly care for children suffering from emotional and psychosocial developmental problems and leads to frequent referrals to other health care professionals. Fragmentation of care ensues and the children who most need the pediatrician are referred elsewhere. The volume-based reimbursement model is also responsible, in part, for underdevelopment of additional domains that can diversify and enrich the career of a pediatrician and decrease burnout – research, teaching, and management.

### Developing the collaborative model of care in community pediatrics

- The second day of the workshop opened with a session on integrated child healthcare. Prof. Mitch Blair from Imperial college in London explained that integration addresses false divisions between clinical and social pediatrics, treatment and prevention, hospital and community. Prof Blair presented some of the conclusions of the Models of Child Health Appraised (MOCHA) study [20]. Primary care needs to adapt continually to changing child health morbidities. Children with psychosocial and behavioral problems generally require community-based multidisciplinary care and the involvement of multiple agencies. Coordination of these by community-based practices must become the norm. He also presented the general practice hub in Imperial as an example of integrated care [21].

Dr. Shoshy Goldberg, National Head Nurse in the MOH discussed the nurse’s role in childcare in the community sphere. She highlighted the need to empower and strengthen the role of specialized nurse in community childcare. Coordination of care, specifically for children with complex conditions, and health promotion are examples for nurse’s involvement. Dr. Goldberg also updated the audience on the Tipot Halav project -positioning MCHCs (Tipot Halav) at the core of services for treating and supporting young families. Tipot Halav should deliver high quality health-promoting counseling and serve as a parenting guidance center.

Prof. Eitan Kerem, the chairman of Goshen, discussed the Goshen initiative. Goshen’s mission is to promote the health and wellbeing of all children within their families in the community, by enhancing the use and dissemination of evidence-based knowledge and practice; advocating for multidisciplinary service delivery and supporting parents in collaboration with all community services. The areas of activity include training, disseminating evidence-based information, developing community programs, performing research, and advocacy. Prof. Kerem concluded by saying that empowerment and integration of existing infrastructures, such as pediatricians, community clinics, Tipat halav, and professional nurses, will improve the care of our children, starting in early childhood.

The last speaker, Sima Hadad from the Israel National Council for the Young Child, spoke about education and health. The focus of her lecture was the need to raise the awareness of community pediatricians to the different stakeholders, beyond the health sector, in order to optimize integrated care. She presented models of successful collaboration between the education and health systems that led to increasing vaccination coverage and health promotion programs, as just two examples.

The session ended with a panel on integrated child healthcare. Prof. Francis Mimouni, Chairman of the National Council for Pediatrics and Child Health, moderated, and Dr. Hadar Yardeni from the MOH, Dr. Stephen Reingold from Meuhedet and Dr. Arie Bahir from Clalit participated. Prof. Mimouni presented the council as an example of a model of integrated care, reflected by the variety of both the members as well as the topics discussed in the council. Dr. Reingold spoke on the integrated care delivered to children under the roof of maternal and child health clinics belonging to municipalities, involving social services and other stakeholders. Dr. Bahir presented Clalit child health centers that include staff of social workers, nurses, dieticians, pharmacists and secretaries who know the families, as an example of a recommended setup for the delivery of successful integrated care to children.

### Recommendations to health funds and the MOH

**Fig. 1:**
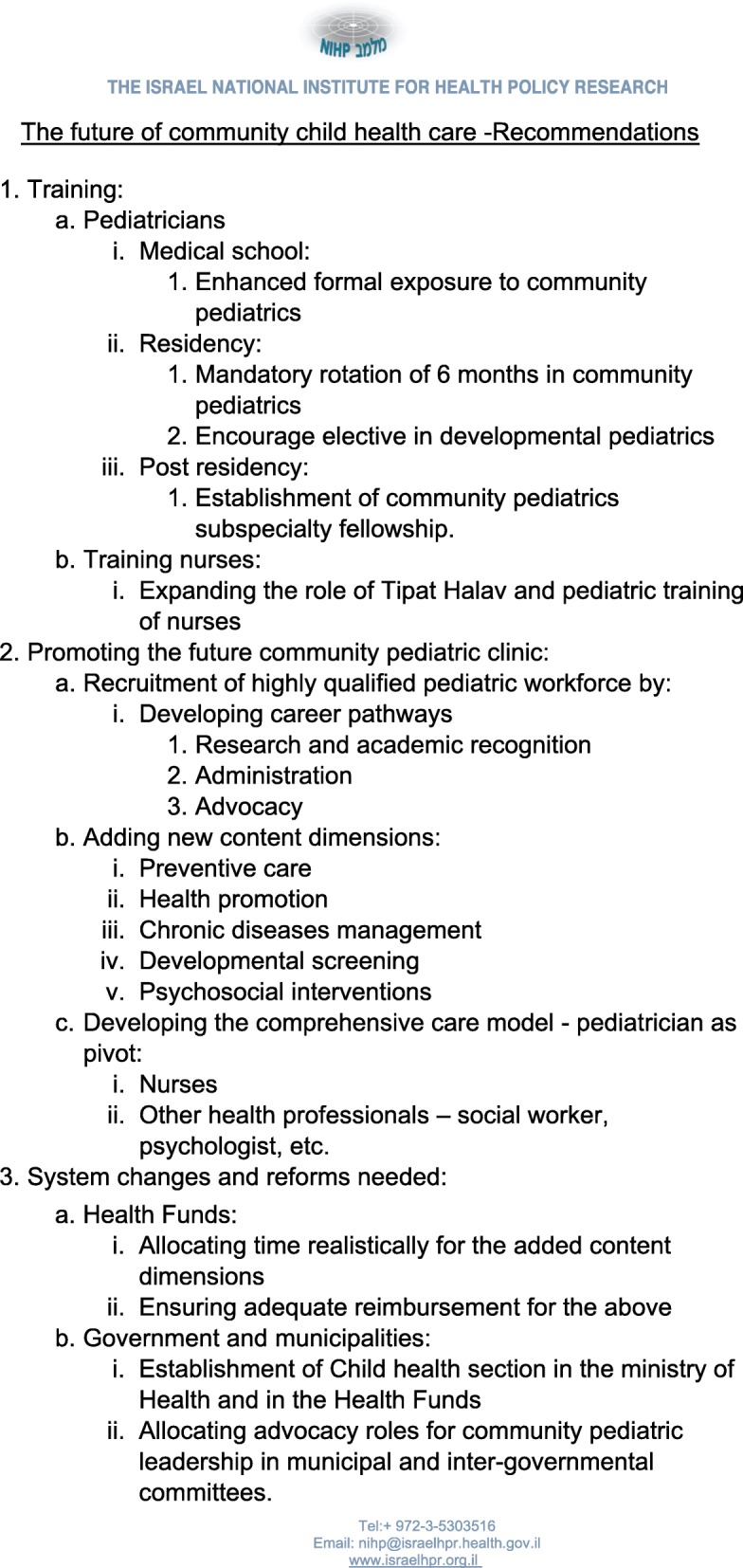
Recommendations to Health Funds and the MOH

Recommendations developed by the organizing committee were presented at the end of the workshop (Fig. 1). A panel of all four Chief Executive Officers (CEOs) of the Israeli Health Funds - Prof. Ehud Davidson (Clalit), Ran Sa’ar (Maccabi), Sigal Regev Rosenberg (Meuhedet) and Nissim Alon (Leumit) concluded the conference. In this panel, all four CEOs, agreed to allocate budget for obligatory block rotations in community pediatrics for all pediatric residents in Israel. This groundbreaking declaration, if fulfilled, has the power to change the attitude of residents towards community pediatrics, increase its attractiveness and eventually lead to the systemic changes needed to provide more appropriate, better and integrative child healthcare.

In conclusion, this national workshop on the future of community child healthcare both raised questions and developed recommendations for policy makers in the MOH and in the Health Funds to improve the care of children in Israel. It is hoped that this effort will lead to steps that will lead to changes in the community healthcare delivery to children in Israel, eventually culminating in better child health.

## Data Availability

The datasets used and/or analyzed during the current study are available from the corresponding author on reasonable request.

## Abbreviations

AAP: American Academy of Pediatrics
ADHD: Attention Deficit Hyperactivity Disorder
ASQ: Ages and Stages Questionnaire
CD: Child Development
CDI: Child Development Institutes
CEO: Chief Executive Officers
CHS: Clalit Health Services
MH: Mental Health
MOCHA: Models of Child Health Appraised
MOH: Ministry of Health
NIHPR: National Institute for Health Policy Research
PCPs: Primary Care Pediatricians
PROS: Pediatric Research in Office Setting
